# Functionalizing the Electrical Properties of Kombucha Zoogleal Mats for Biosensing Applications

**DOI:** 10.1021/acsomega.4c01227

**Published:** 2024-07-08

**Authors:** Anna Nikolaidou, Alessandro Chiolerio, Mohammad Mahdi Dehshibi, Andrew Adamatzky

**Affiliations:** † Unconventional Computing Laboratory and Department of Architecture and Environment, University of the West of England, Bristol, BS16 1QY, United Kingdom; ‡ Bioinspired Soft Robotics, Istituto Italiano di Tecnologia, Via Morego 30, 16163 Genova, Italy; ¶ Escuela Politécnica Superior, Departamento de Informática, Universidad Carlos III de Madrid, Leganés, 28911, Spain; § Unconventional Computing Laboratory, University of the West of England, Bristol, BS16 1QY, United Kingdom

## Abstract

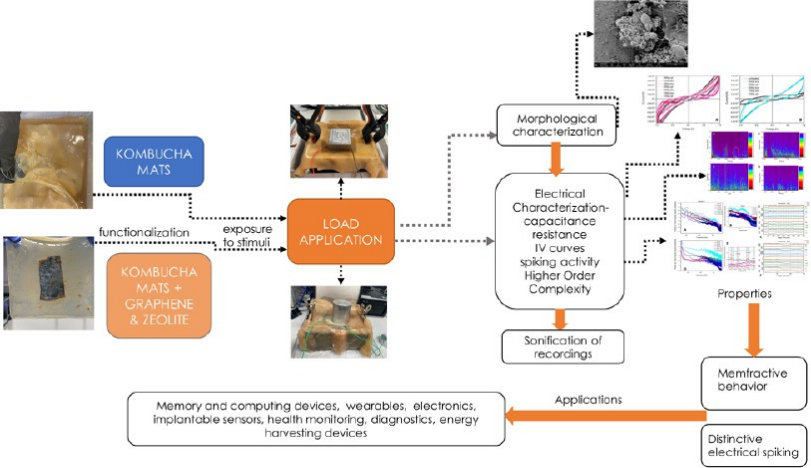

Kombucha is a type of tea that is fermented using yeast and bacteria. During this process, a film made of cellulose is produced. This film has unique properties such as biodegradability, flexibility, shape conformability, and ability to self-grow as well as be produced across customized scales. In our previous studies, we demonstrated that Kombucha mats exhibit electrical activity represented by spikes of the electrical potential. We propose using microbial fermentation as a method for *in situ* functionalization to modulate the electroactive nature of Kombucha cellulose mats, where graphene and zeolite were used for the functionalization. We subjected the pure and functionalized Kombucha mats to mechanical stimulation by applying different weights and geometries. Our experiments demonstrated that Kombucha mats functionalized with graphene and zeolite exhibit memfractive properties and respond to load by producing distinctive spiking patterns. Our findings present incredible opportunities for the *in situ* development of functionalized hybrid materials with sensing, computing, and memory capabilities. These materials can self-assemble and self-grow after they fuse their living and synthetic components. This study contributes to an emergent area of research on bioelectronic sensing and hybrid living materials, opening up exciting opportunities for use in smart wearables, diagnostics, health monitoring, and energy harvesting applications.

## Introduction

There has been a significant increase in the use of cellulose as an alternative sustainable and renewable material in various fields such as medicine, packaging, food, and textiles. Cellulose is a natural biodegradable polymer and is considered to be one of the most abundant macromolecules in nature. It is produced by a wide range of living organisms, from bacteria to plants.^1−3^ Bacterial cellulose is known to exhibit a higher purity, mechanical strength, water-holding capacity, chemical stability, and biological adaptability than cellulose produced by plants. This is due to a lack of lignin and hemicellulose polymers.^4^ Acetic acid bacteria produce acetic acid by metabolizing carbon sources into acetic acid and ethanol through their oxidative fermentation pathways.^5^ They are excellent producers of cellulose,^6^ which can be obtained in a very short time.^7,8^ Cellulose is organized in hydrogels and features interesting trains of spikes of extracellular electrical potential.^9^ These potential spikes could be used to enable reactive sensing wearables by means of living colonies of bacteria.^10^ When a community of bacteria, such as acetic acid bacteria and yeast, form a symbiotic relationship, they can enhance cellulose production through their combined microbial metabolism.^4^ This synergy is found in Kombucha cellulose, which emerges during the fermentation of Kombucha tea. Kombucha tea is a popular probiotic beverage that is made by fermenting sugared tea with a SCOBY, a symbiotic culture of over 20 species of bacteria and yeast.^11−13^ During fermentation, the microorganisms consume sucrose as the primary carbon source, while tea extract provides the nitrogen source. In the presence of oxygen, the SCOBY produces organic acids, carbon dioxide, and a floating biofilm composed of cellulose. The thickness of the film varies depending on the nutrients and breeding time.^14^ Kombucha cellulose shows high biocompatibility due to its enhanced cell-matrix interactions, cell signaling pathways, and ability to maintain cellular homeostasis.^15^ In our previous studies, we demonstrated that Kombucha cellulose mats show rich dynamics of electrical activity, represented by spikes of electrical potential. This makes them an exciting material for developing living bioelectronic materials with active electrical properties, sensing and computing capabilities.^16,17^ The electroactive nature of the cellulose mats also makes them suitable for biosensing applications.

Live Kombucha cellulose mats possess various chemical, electrical, and mechanical properties. They have a high production rate of cellulose, are cost-effective and flexible, and can take customized shapes while retaining the ability to self-grow. These mats can be produced in different sizes and thicknesses, making them ideal materials for scaffold production. They can be used to create biocomposites with targeted and enhanced functionalities suitable for applications such as smart wearables, soft robotics, and building materials.

To enhance the electrical properties and sensing capabilities of Kombucha cellulose mats, in this study we combine graphene and zeolite nanoparticles for the functionalization of the mats. The selection of zeolite-graphene nanoparticles as cell carriers within the Kombucha zoogleal mat was based on their distinct, but collaborative characteristics,^18−20^ leading to the improvement of the mat’s electrical conductivity, sensitivity, and stability. Graphene is a two-dimensional carbon nanomaterial with excellent electrical conductivity, mechanical strength, high thermal conductivity, and high electron mobility at room temperature.^21−26^ These exceptional characteristics qualify graphene for the reinforcement of biopolymer matrixes.^27^ The integration of graphene into the cellulose matrix enables the formation of electrical conductive pathways, facilitating effective electron transfer and thus improving the overall electrical conductivity of the material.^28,29^ Additionally, Kombucha cellulose can strongly bind with graphene because of the many hydroxyl groups in the cellulose units, allowing for effective interactions between the cellulose and graphene such as hydrogen bonding.^27,30−33^ Graphene is therefore an ideal candidate for functionalization and enhancement of the electrical properties of the Kombucha cellulose mats. Zeolites are a class of aluminosilicates with a crystalline lattice structure and pores of molecular dimensions.^34−39^ They have been used for various applications including zeolite-based biosensors. Zeolite-based biosensors with different characteristics can be obtained by varying their properties such as ion exchange behavior, particle size, surface groups and pore size.^40^ They have also been used for improving the performance of existing sensors due to their absorption, diffusion and catalytic properties.^41^ Equal to graphene, zeolites present a multitude of benefits for functionalization. Due to their structure, they have a large surface area, high adsorption capacity, and high selectivity, enabling the absorption of various components.^37,42−44^ Consequently, they can provide an expanded surface area which facilitates particle and cell immobilization and attachment. Additionally, zeolites facilitate a suitable environment for encapsulation, enabling immobilized biomolecules to have enhanced stability,^45^ ensuring the material’s durability and functionality. Taking advantage of the above complementary properties of zeolite and graphene nanoparticles, we aim to use them as carriers for integrated cells. Graphene contributes to exceptional electrical conductivity and mechanical strength, whereas the zeolite nanoparticles provide an expanded surface area to facilitate immobilization, adherence, and improved stability. Their unique combination into the cellulose fibers of the Kombucha mat allows for enhanced sensitivity, stability, and electrical responses to mechanical pressure.

This study presents new insights into the *in situ* fictionalization of Kombucha pellicles to enhance their electrical properties and introduce targeted responsive capabilities. We performed electrical measurements on both unmodified and functionalized cellulose mats under mechanical stimuli using weight load application and pattern projection. The results showed that the functionalized Kombucha cellulose mats exhibit significantly higher sensitivity to external input compared to the control unmodified cellulose mats. This work contributes to the emerging field of bioelectronic sensing and establishes Kombucha cellulose as an exciting, sustainable scaffold material for information recognition and transmission.

## Methods

### Preparation of Pure and Functionalized Kombucha Cellulose

For modulating the electrochemical properties of Kombucha cellulosic mats, graphene and zeolite were used for *in situ* functionalization. For the production of the unmodified and functionalized Kombucha cellulose samples, a SCOBY culture of yeast and bacteria acquired from Freshly Fermented, Ltd. (Lee-on-the-Solent, PO13 9FU, UK) was inoculated in a sucrose tea infusion. For the infusion, black tea was selected as it has demonstrated higher levels of cellulose production.^4^ The infusion was prepared with 5 L of boiled tap water, 500 g of white granulated sugar (Tate & Lyle, UK), and 10 black tea bags (Taylors Yorkshire Teabags, UK, 125 g) in a plastic container and left to reach room temperature (22 °C). The SCOBY mat was then placed in the solution, and the container was stored in darkness and maintained at static conditions of 19 °C. A lid with 8 × 0.5 mm^2^ holes was used to fully enclose the plastic container. Film formation was observed on the sixth day as thin layers above and below the native SCOBY, and the thickness increased with the fermentation time. We replaced the solution every 12–14 days before the increase in cellulose production reached a plateau, following the same infusion protocol as that mentioned above. Our rationale was that a rapid decrease of sucrose has been reported from day 5 to day 15, which is attributed to the yeast extracellular enzyme production^4^ resulting from the kinetic behavior that the Kombucha SCOBY exhibits, where yeasts hydrolyze sucrose for their own consumption, producing ethanol and carbon dioxide, and making available reducing sugars for bacterial metabolism.^11,46^ After 8 weeks, the cellulose mats were transferred to a 240 × 240 mm^2^ Petri dish each. 30 mL of newly made sucrose black tea solution was added to each Petri dish, instigating the fermentation process and allowing for the continuation of mat growth. After 7 days of inoculation, we proceeded with the experiments.

The two cellulose mats exhibited a high intensity of yellow and brown color. The yellowness can be attributed to the Maillard reactions taking place during the fermentation and film conditioning occurring between the sugars and amino acids in addition to colored phytochemicals.^4^ The brown color has been reported to be caused by melanoidins, which are colored and nitrogen-containing compounds.^47,48^ Some inhomogeneity observed in the visual appearance of the functionalized Kombucha mat is a typical feature of materials produced by microorganisms. Kombucha zoogleal mats are multispecies systems synthesized by variable natural symbiotes, yeast and bacteria. During the process of fermentation, cooperative and competitive interactions occur among the microbes. The nonuniformity observed in the growth of the modified Kombucha mat can be attributed to their ongoing interactions and metabolic activities. Additionally, the surface adherence properties of the mat can be directly affected by operational conditions such as the pH, temperature and sugar concentrations.^49,50^ These factors account for the nonuniformity observed in the mat, which grows as multiple layers with strands suspended down. The inhomogeneity in growth may impact the experimental phenomena; however, these potential variations in the density, size, and porosity of the cellulose pellicles can enhance the distinct functions of the material, leading to tailored and controlled properties such as interactions with the functional nanomaterials (graphene and zeolite) and favorable mechanical properties. Microstructural characterization such as scanning electron microscopy and Fourier transformed infrared spectroscopy analysis as well as further analysis of the mat’s properties via tensile tests and water vapor permeability could offer valuable information on correlations between inhomogeneity in growth, composition, and variations in the structure.^51^ The thickness of the mats was 3.3 and 4.2 mm and was measured with an electronic digital caliper (accuracy ±0.2 mm, Vodlbov, China). The pH was monitored starting from 6.49 and reducing to 3.02 using a digital pH meter (accuracy ±0.1% ± 2 digits, VWR pH110, Belgium).

**Figure 1:**
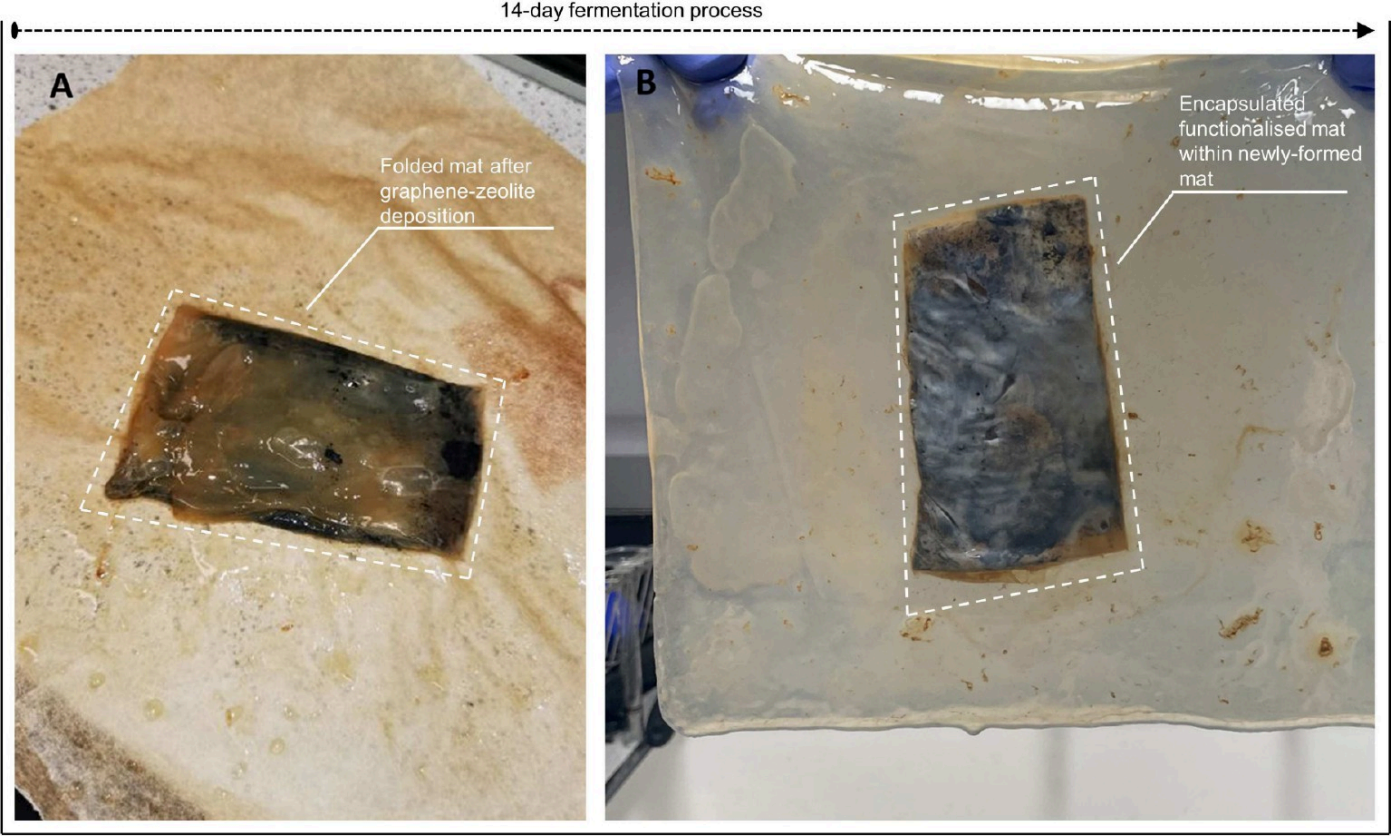
In situ preparation process of functionalized Kombucha mat. (A) The Kombucha cellulose mat after functionalization with graphene and zeolite Y nanoparticles. After homogeneous deposition, the mat was folded to avoid spillage of the mixture when entered back to the Kombucha solution. (B) Functionalized Kombucha cellulose mat after 14 days of growth.

One cellulose mat sample (thickness 3.3 mm) was functionalized with graphene and zeolite. For the functionalization, 5.372 g of graphene powder (HCG10000-P-1, Graphitene Energy Storage Series, https://www.graphitene.com) were mixed with 1.289 g of Zeolite Y powder (ThermoFisher Scientific UK, 045862.36). The thickness of graphene powder was 1–3 layers with a lateral size 0.5–5 μm, a surface area of 500 m^2^/g, and an electrical conductivity >104 S/m. The surface area of zeolite Y was 927 m^2^/g. To achieve good interfacial bonding between the graphene and zeolite molecules, we used PTFE solvent (Sigma-Aldrich, 665880), and the mixture was stirred until a homogeneous solution was achieved (the total mixture weight was 12.965 g). The graphene-zeolite Y mixture was deposited on the surface of the Kombucha cellulose film using a razor blade and dispersed homogeneously until it was fully coated. After coating the top surface, the film was folded in such a manner that the mixture remained enclosed within the film. For the in situ fabrication of the Kombucha-graphene-zeolite hybrid living material, the method of microbial fermentation was used and therefore, the functionalized film was placed back again into the Petri dish to allow further growth. After 14 days, a new cellulose mat was formed, fully encapsulating the functionalized cellulose mat (Figure 1). The size of the two samples was 240 × 240 mm^2^.

### Experimental Setup and Characterization

**Figure 2:**
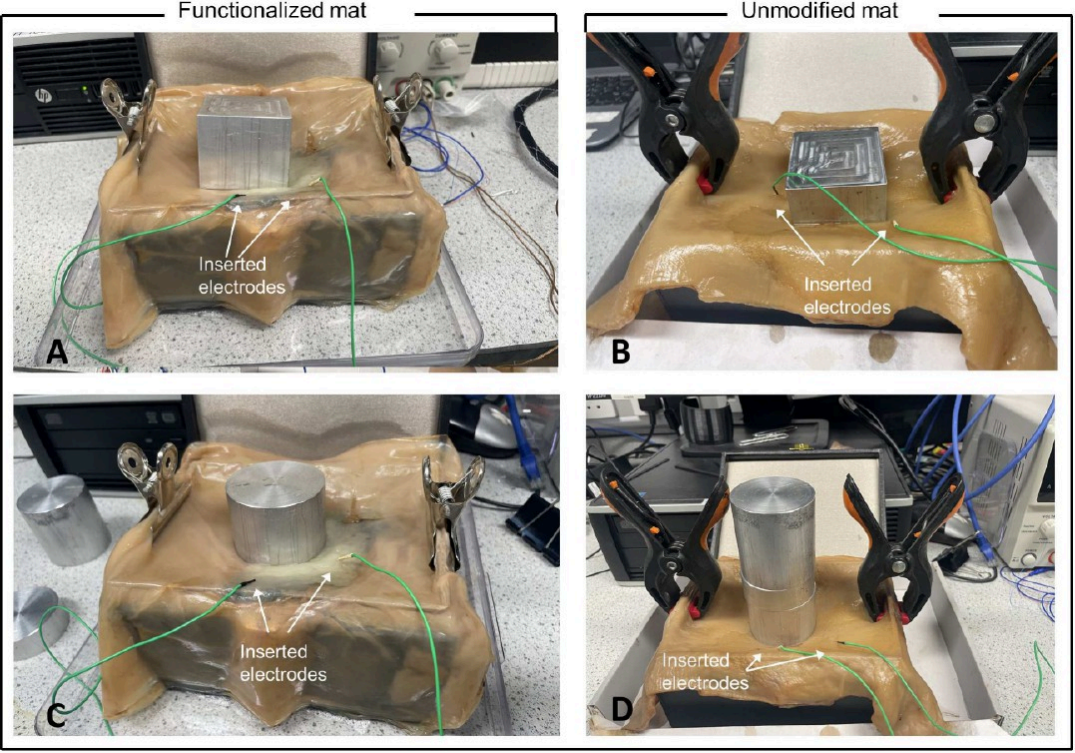
Electrical characterization of unmodified and modified Kombucha mats. A pair of iridium-coated stainless-steel subdermal needle electrodes was inserted into the mats for the performance of measurements. (A), (C) Impedance, capacitance, resistance, and *I*–*V* measurements taken from functionalized Kombucha cellulose mat, 240 × 240 mm^2^, under square and circular weights of 100, 200, 300, and 600 g. (B), (D) Impedance, capacitance, resistance, and *I*–*V* measurements taken from functionalized Kombucha cellulose mat, 240 × 240 mm^2^, under square and circular weights of 100, 200, 300, and 600 g.

The unmodified and functionalized Kombucha cellulose mats were placed on top of a cardboard box with dimensions of *W* × *L* = 122 × 160 mm^2^ and secured in place with plastic and metallic clamps prior to performing any measurements. To assess the response and mechanical properties of the samples when exposed to compression, we used a load application. For the mechanical stimulation of the samples, manually cut square aluminum weights of 100 g (*W* × *L* × *H* = 51 ×51 × 14.2 mm^3^), 200 g (*W* × *L* × *H* = 51 × 51 × 28.4 mm^3^), and 300 g (*W* × *L* × *H* = 51 × 51 × 42.7 mm^3^) and circular aluminum weights of 100 g (*D* × *H* = 51 × 18.1 mm^2^), 200 g (*D* × *H* = 51 × 36 mm^2^), and 300 g (**D*×*H*=* 51 × 54.4 mm^2^) were employed. The weights were applied to both samples in different combinations to attain loads of 100, 200, 300, 400, and 600 g (Figure 2). Both patterns of weights (square and circular) were applied to the two cellulose samples.

Electrical characterization of the unmodified and functionalized mats prior to and during the application of 100, 200, 300, and 600 g loads was performed. A pair of iridium-coated stainless-steel subdermal needle electrodes (Spes Medica S.r.l., Italy), with twisted cables, was pierced through the mats with a distance between the electrodes of 50–60 mm. A Keithley 2450 SourceMeter was used for the provision of high-precision current–voltage (*I*–*V*) sweep measurements (type linear dual, voltage range from −1 to 1 V, step 10 mV, count finite 20, source limit 1A). Impedance, capacitance, and resistance measurements were taken using a BK Precision LCR Meter (model 891) within the frequency range of 20 Hz and 300 kHz. The experiments were repeated to ensure error elimination.

**Figure 3:**
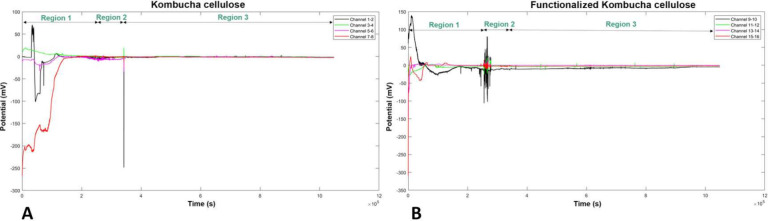
Spiking activity of 240 × 240 mm^2^ samples when exposed to different loads. The graphs display the electrical activity over time, measured in potential (mV) versus time (sec), from both Kombucha (Ch1–Ch4) and Kombucha with graphene-zeolite Y cellulose mats (Ch5–Ch8). In (A), Region 1 shows the application of a 100 g box load, Region 2 represents the application of a 400 g box load, and Region 3 represents the application of a 300 g cylinder load. In (B), Region 1 represents the application of a 100 g cylinder load, Region 2 represents the application of a 400 g cylinder load, and Region 3 represents the application of a 300 g box load.

For the recordings of the electrical activity before and after the weight application, an ADC-24 (Pico Technology, UK) high-resolution data logger with a 24bit A/D converter was used. Weights of square and circular patterns of 100, 300, and 400 g were applied to the samples. Eight pairs of iridium-coated stainless-steel subdermal needle electrodes were inserted in the samples; the first 4 (channels 1–2, 3–4, 5–6, and 7–8) in the unmodified cellulose sample and the remaining 4 (channels 9–10, 11–12, 13–14, and 15–16) in the functionalized sample (Figure 3). We recorded electrical activity simultaneously, one sample per second, and each pair of electrodes reported a difference in the electrical potential between the electrodes. All readings were taken at a room temperature of 19 °C. During the recording, the logger undertook as many measurements as possible (typically up to 600 per second) and saved the average value. The distance between the electrodes was 10–20 mm.

The microstructure of the unmodified and functionalized Kombucha cellulose samples was analyzed by scanning electron microscopy and environmental scanning electron microscopy (FEI Quanta 650). For the preparation prior to imaging, the samples were air-dried and coated with a gold layer using an Emscope SC500 gold sputter coating unit. Images were acquired with a magnification range of 1300–20000× and a high voltage of 2 kV. To the best of our knowledge, this is the first time that Kombucha cellulose has been functionalized to evaluate its electrical properties and response to load application.

## Results

### Morphological and Chemical Characterization

**Figure 4:**
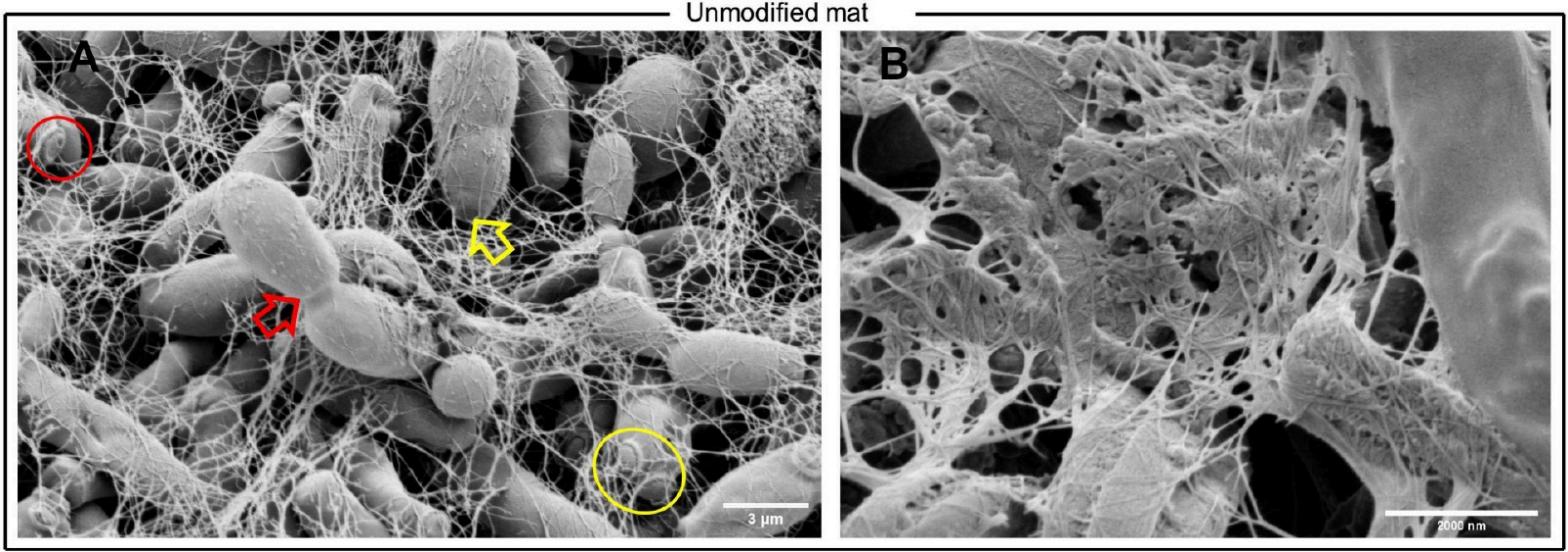
SEM images of the pure Kombucha mat. A) The presence of multiple-size yeast cells is identified, with lengths ranging from 1.93 to 7.76 μm. Cell bud scars, birth scars, multipolar budding, and bud–parent sites are marked in the image with red circles, yellow and red arrows, and a yellow circle accordingly. Image magnification is 8000×. B) Network of cellulosic fibers with a branching structure. Image magnification is 20000×.

SEM imaging performed on pure and functionalized Kombucha-graphene-zeolite samples demonstrated that the structure of the pure Kombucha mat changes significantly with the integration of the graphene-zeolite particles. The morphological features of pure Kombucha reveal that the microbial community of yeast and bacterial cells present mostly ellipsoidal structures, are compactly and uniformly dispersed, and are entangled into a dense mesh of cellulosic fibers that form three-dimensional web-like structures. The presence of multiple-size yeast cells is observed, spanning from 1.93 to 7.76 μm in length. Chain yeast elongations are visible, and protrusions on the cell surfaces show cell budding mechanisms.^52^Figure 4 shows bud and birth scars as well as bud–parent junctions (marked with red circles and yellow and red arrows accordingly). Multipolar budding is also clearly identified on some cells (marked with a yellow circle).

**Figure 5:**
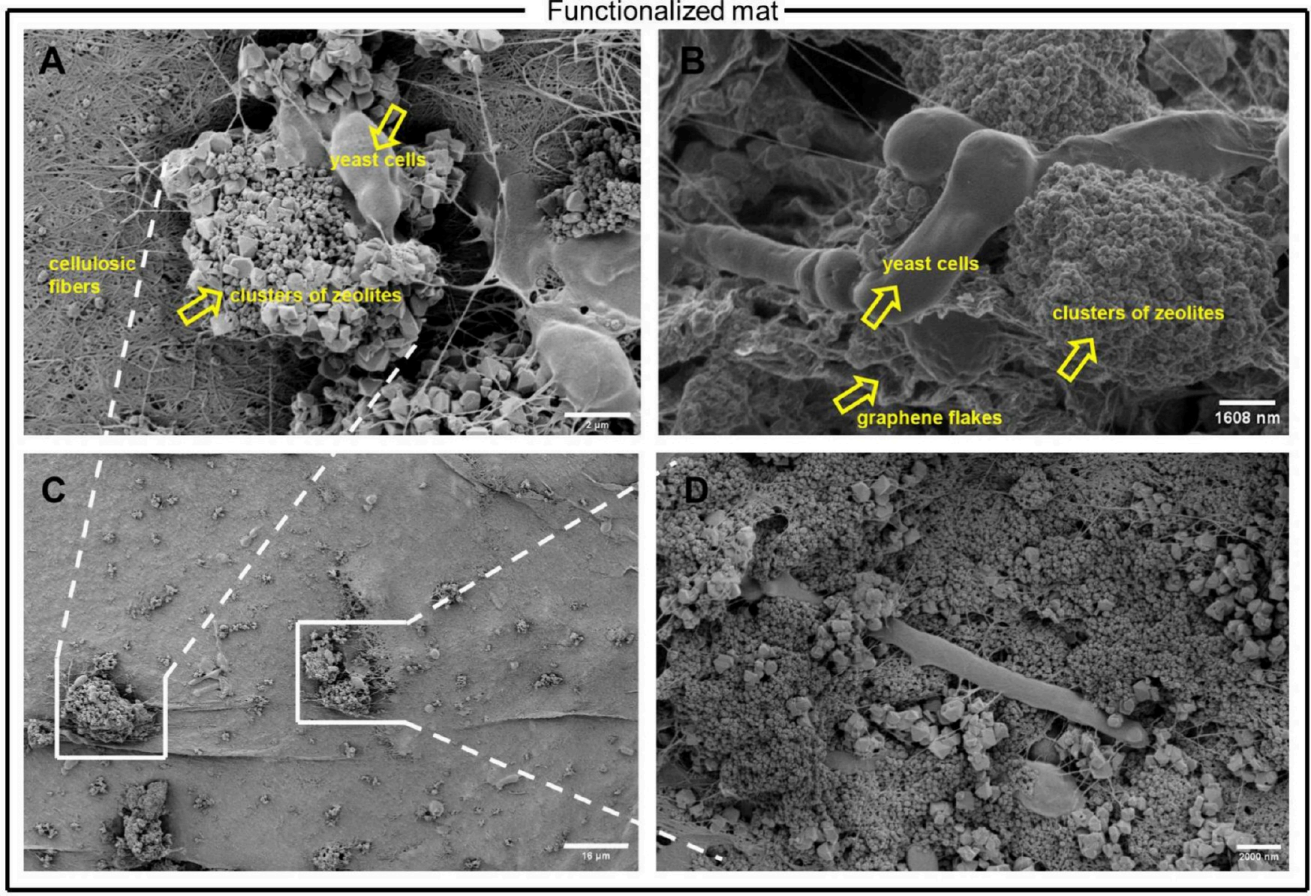
SEM images of functionalized Kombucha mat. A) Clusters of zeolite crystals entangled with microbes. Image magnification is 10000×. B) Graphene-zeolite assembly formations where graphene flakes attached to zeolite crystals and yeast cells are obsreved. Image magnification is 10000×. C) Lower magnification image showing distribution of nanoparticles. Image magnification is 1300×. D) Yeast cells integrated into zeolite-graphene nanoparticles. Image magnification is 7000×.

Images of the functionalized sample revealed dense aggregations of zeolite crystals with morphologies of spherical agglomerates of tiny crystals next to larger polyhedrons surrounded by typical flakes of graphene particles^53^ and integrated with scattered Kombucha microbes. The presented high-density configurations may be attributed to the high microporosity of the zeolite active sites, which provides space for the adsorption and exchange of cations, leading to the attachment of the graphene molecules. This connection could favor electron transport and contribute to the electrochemical performance of the functionalized Kombucha film. In addition to this, the cellulose fiber network appears to be less dense and finer, where the graphene-zeolite formations are present. The measured cluster assemblies have diameters of 9.61 and 7.75 μm (see Figure 5). Contrary to pure Kombucha images, microbial cells appear in lower densities in the functionalized Kombucha images, but elongations and birth scars are still present, suggesting that growth and replication mechanisms continue to take place after functionalization. The above findings indicate that the new structural formations may involve the microscopic movement of the microbial population due to the mechanical and physical forces built up from the graphene-zeolite encapsulation during in situ functionalization. Moreover, it is observed that the graphene-zeolite particles are found entangled with and attached to the microbial cells and in parts enveloped by the cells, suggesting the integration of the living biological and synthetic components, therefore indicating the possible synthetic morphogenesis of a new and higher ordered structure. This may open up remarkable opportunities for the in situ development of functionalized hybrid materials that can self-assemble, self-replicate, self-generate, and self-grow after the fusion of their living and synthetic components.

**Figure 6:**
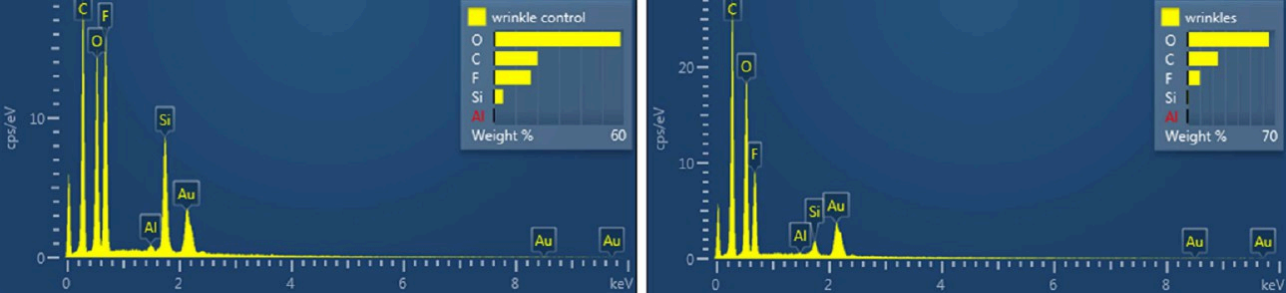
Energy-dispersive spectroscopy (EDS) graphs of two samples collected from different sites of the functionalized Kombucha mat, showing the distribution of elements. The graphs reveal the presence of silicon (Si), aluminum (Al), and carbon (C) peaks, indicating the integration of the zeolite and graphene nanoparticles within the Kombucha mat. Additionally, the presence of fluorine (F) is observed due to the use of PTFE solvent to achieve good interfacial bonding between the graphene and zeolite molecules. The gold (Au) peaks are attributed to the gold coating applied to the sample to increase its conductivity for improved scanning electron microscopy imaging. The peaks of elements in both EDS graphs follow a similar trend, indicating a homogeneous distribution of the graphene and zeolite nanoparticles in both samples and therefore, incorporation within the Kombucha matrix.

Energy-dispersive spectroscopy (EDS) shows the distribution of elements within the functionalized Kombucha film (Figure 6). In the material distribution analysis, very low Al content is observed, demonstrating high hydrothermal stability.^54^

### Direct Current and Impedance Properties

**Figure 7:**
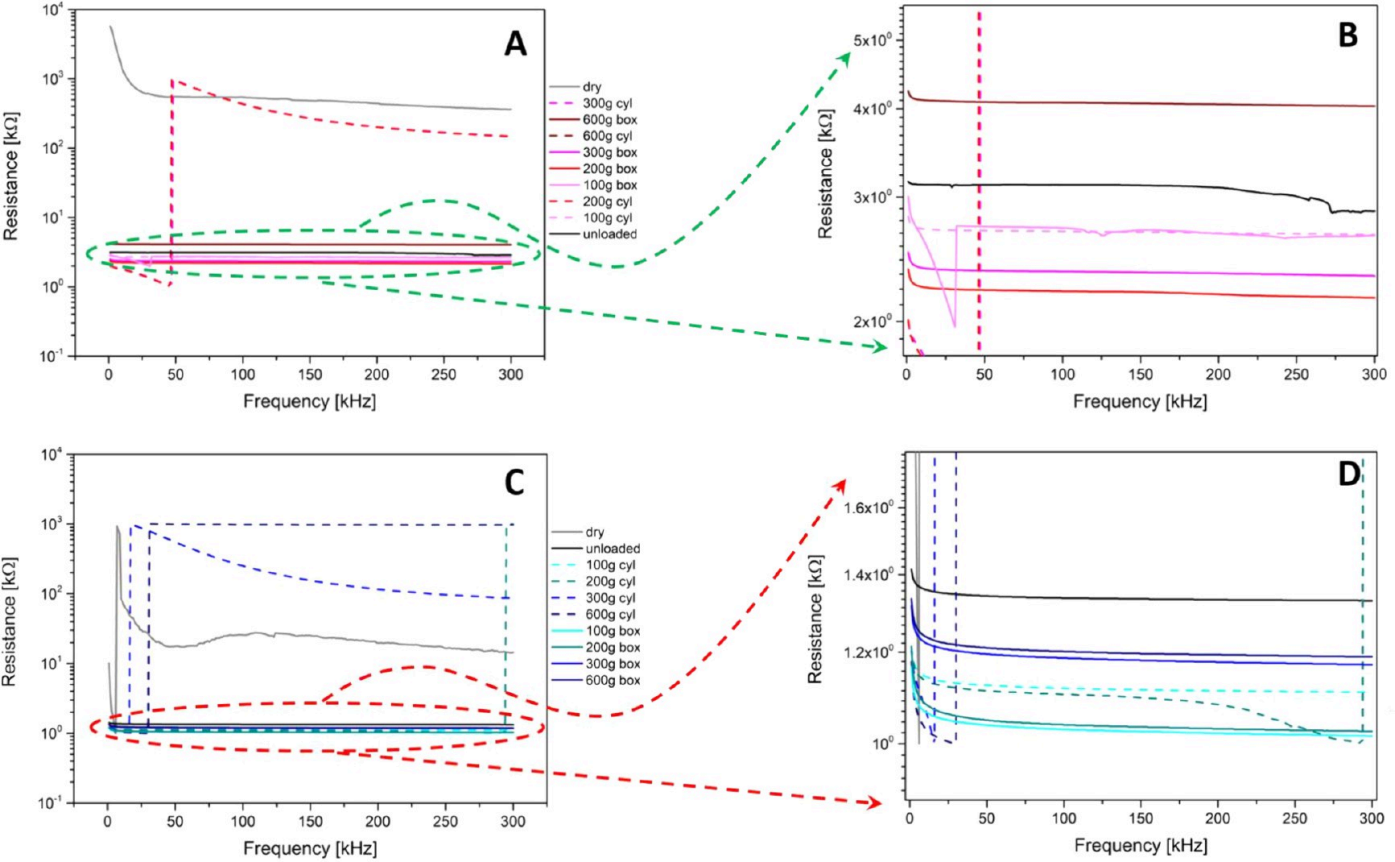
Series resistance as a function of the frequency. (A) Kombucha response to the different loads and (B) inset showing a zoomed-in view of the circled area. (C) The functionalized Kombucha response to the different loads and (D) inset showing a zoom of the circled area.

Measurements were carried out in the frequency range between 1 and 300 kHz. Series resistance as a function of the frequency is shown in Figure 7. The upper row shows the Kombucha mat in different conditions (dry, wet, and unloaded, and with cylinder and box loads from 100 to 600 g), while the bottom row shows functionalized mats. The dry condition is such that the functionalized mat features a resistance 1 order of magnitude lower than the pristine Kombucha mat due to the enhancement provided by graphene addition. The consecutive loading of the mat produces a reduction of the resistance that achieves a maximum with the intermediate loading of 200 g and then recovers back and eventually becomes even higher than the unloaded measure for the loading of 600 g. This effect might be due to pseudoelastic stretching of the matrix under the normal forces exerted by the loads with concurrent reduction of the section available for conduction. A remarkably similar trend might be found in the functionalized Kombucha mat, where the 100 and 200 g loads reduce the resistance and the 300 and 600 g loads increase it, to values lower than the unloaded case. Regarding the shape of the load, we note that the cylinder weight can produce very pronounced responses, particularly in the load range of 200–300 g for both mats. In Figure 7 we see that under some conditions the pristine Kombucha undergoes a transition and around 50 Hz its resistance increases by 3 orders of magnitude, featuring a response that is pretty similar to that of the dry mat. This happens for the 200 and 300 g cylinders, during a more severe deformation due to the smaller contact area in comparison to the box loads, which are more likely able to expel the extracellular fluids and produce a less conductive state. Similarly, the functionalized Kombucha mat shows a sudden jump in the resistance by 3 orders of magnitude during the application of 300 and 600 g cylinders, producing a response that is even less conductive than the dry mat, probably because of the zeolite dielectric contribution to resistance.

**Figure 8:**
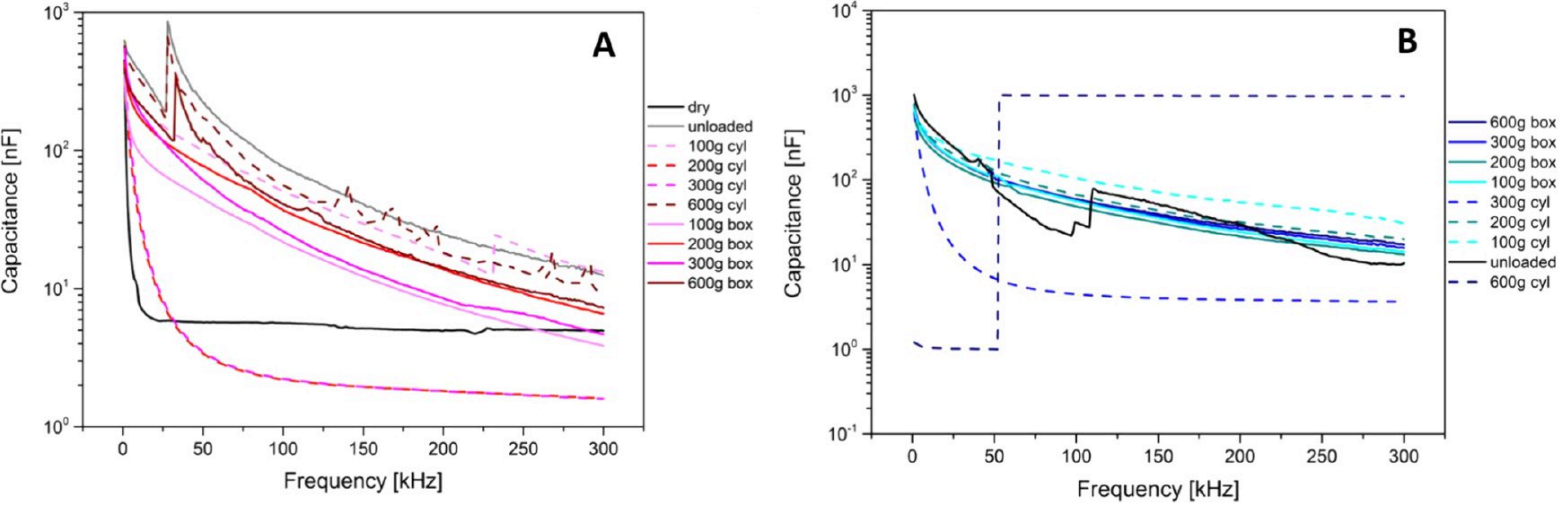
Capacitance as a function of the frequency. (A) Kombucha response to the different loads and (B) functionalized Kombucha response to the different loads.

In Figure 8, the capacitance is shown in the same frequency range as that discussed above. The unloaded curve of the pristine mat versus the functionalized one shows that normally, the capacitance of the latter is approximately double that of the former, which can be due to the addition of zeolite compounds having a higher dielectric constant. Nevertheless, we should remember how much water content in a mat can influence this aspect, as the dielectric constant of water is very high: The dry mat curve shows how small its capacitance can be in the absence of water. Looking at the pristine mat curves, we cannot infer any specific pattern: the measurements cannot correlate with the load amount and/or its shape. On the contrary, the functionalized mat curves feature a more controlled behavior that perfectly maps the observations based on the resistance previously discussed: the deformation produced by 100 and 200 g loads reduces the capacitance, as the displacement of the hydrogel under the force exerted by the load reduces the surface of the dielectric layer incorporated in the capacitor. Other phenomena might occur, such as the capacitive coupling with the load materials (i.e., aluminum and polymers), making it difficult to infer the shape of the load from the measurements. In Figure 8 we can notice some sudden jumps of the capacitance, particularly the pristine Kombucha in dry conditions and after application of 600 g loads (both cylinder and box): Eventually squeezing out of the hydrogel more water by means of the highest loads produces a response that follows that of a dry material.

**Figure 9:**
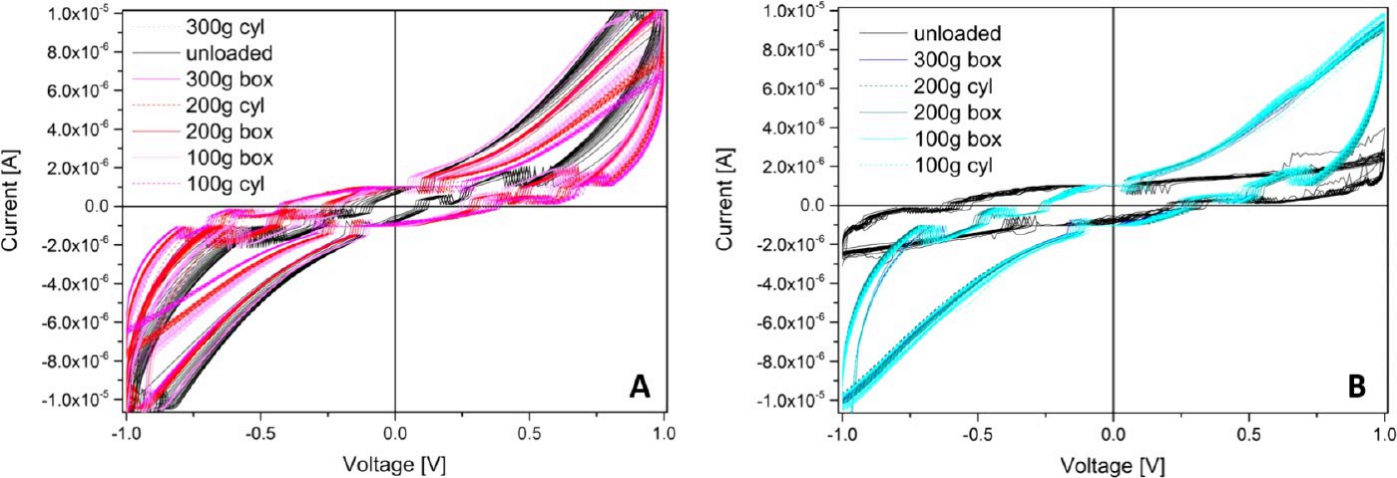
Hysteresis cycles showing the IV response of the mats. (A) Kombucha response to the different loads and (B) functionalized Kombucha response to the different loads.

Another important analysis performed is the collection of *I*–*V* curves along multiple hysteresis cycles to put in evidence typical current ranges and eventually a memristive behavior. Figure 9 shows that in the 1 V range, the currents are on the order of 10 μV for both the pristine and functionalized mats, but in the former case, all curves are very close, and the loading does not provide any strong variation. However, in the latter we can see how much the loaded curves differ from the unloaded one, having currents 5 times bigger. Therefore, loading the functionalized mat produces higher currents (lower resistances), while loading the pristine mat produces lower currents (higher resistances). An enhanced conductivity can explain this effect due to the percolation of the fillers added to the functionalized mat. The higher pressure creates more contact points between inorganic conductive fillers, such as the graphene flakes, and reduces the mat resistance.

### Spiking Activity and Higher Order Complexity Analyses

**Figure 10:**
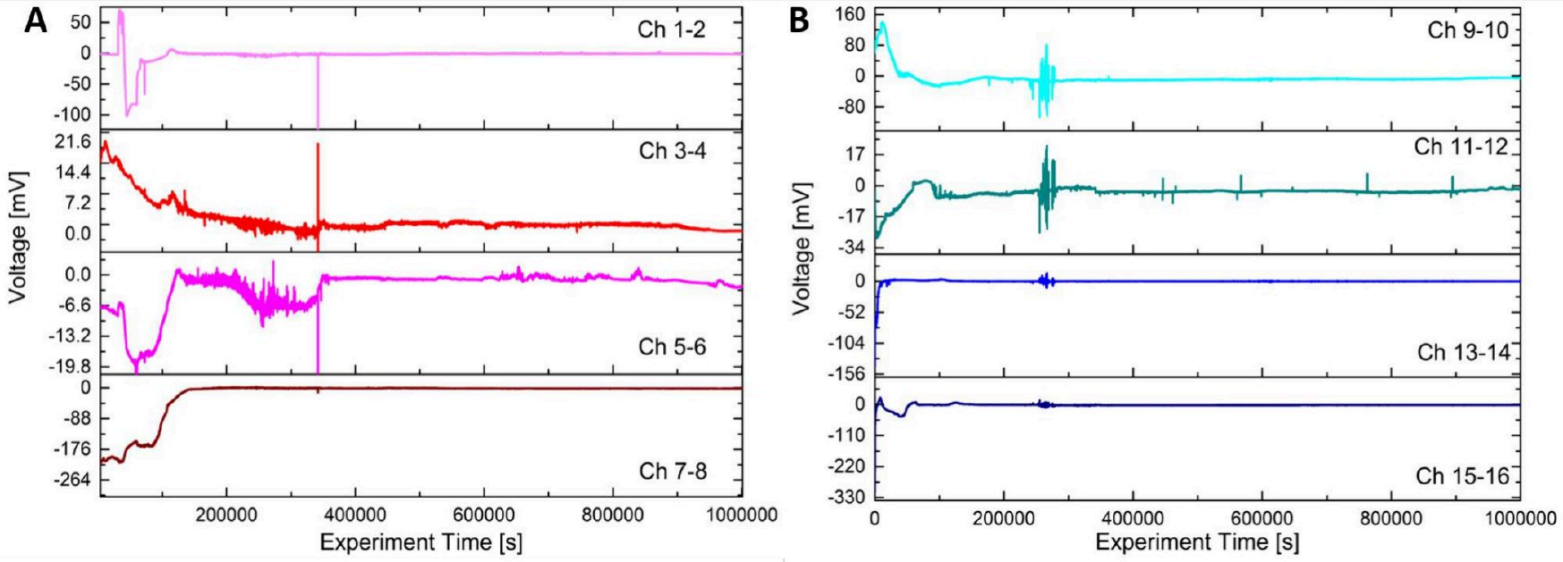
Recordings showing the spiking response of the mats. (A) Kombucha response to the different loads and (B) functionalized Kombucha response to the different loads.

**Figure 11:**
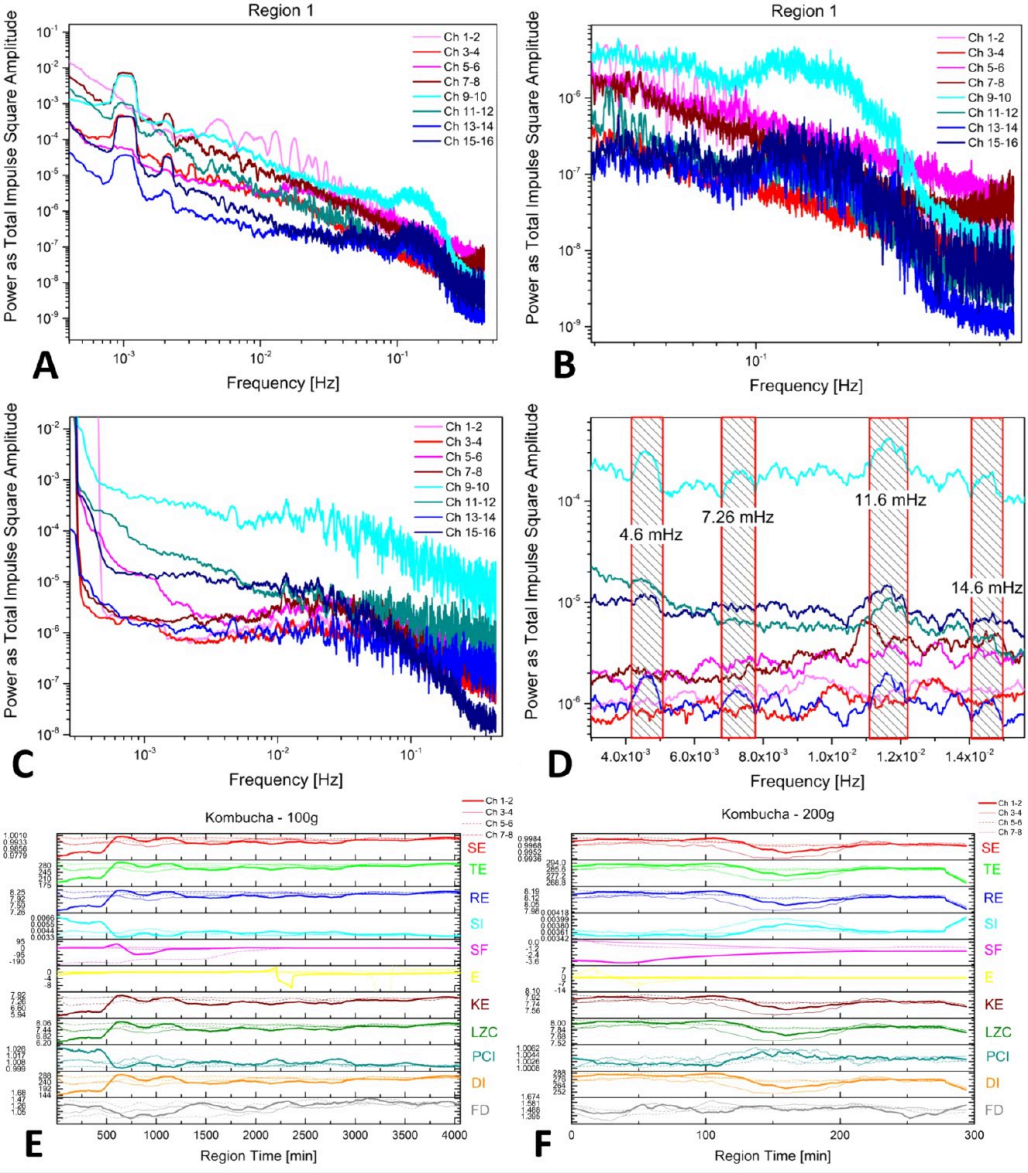
Regional details of the spiking activity features. (A) The fast Fourier transforms correspond to 100 g loading of the Kombucha mat and functionalized Kombucha mat. (B) A zoomed-in view highlights a broad peak structure. (C) The fast Fourier transforms for the region corresponding to 200 g loading of the Kombucha mat and functionalized Kombucha have been shown. (D) A zoomed-in view highlights a broad peak structure. (E) Higher-order complexity measures for the same region have been provided, pristine mat. SE stands for Shannon entropy, TE represents Tsallis entropy, RE stands for Rényi entropy, SI represents Simpson index, SF represents space filling, E represents expressiveness, KE represents Kolmogorov complexity, LZC represents Lempel–Ziv complexity, PCI represents permuted complexity index, DI represents diversity index, and FD represents fractal dimension. (F) Higher-order complexity measures for the same region, pristine mat, have been provided.

The spontaneous spiking activity of two mats was continuously recorded while loads were being applied. The recorded profiles from four differential channels are shown in Figure 10 for the entire measurement period of 11 days. By examining each channel, we can observe a certain degree of correlation involving transitions and increased spiking activity with remarkably similar characteristics. Typically, the spontaneous activity range is around 20 mV. However, some channels exhibit a large baseline fluctuation of about 175 mV (for example, channels 1–2 and 5–6 of the pristine mat) and approximately 200 mV (for example, channels 9–10 of the functionalized mat). To gain a clearer understanding of what is happening in various situations, we used the fast Fourier transform (FFT) and higher order complexity (HOC) metrics^55−57^ as shown in Figure 11.

The FFT analysis shows how the noise spectrum across the mat sensors is distributed over different frequencies, ranging from approximately 1 mHz up to 0.5 Hz, corresponding to half of the sampling frequency. The first row of graphs depicts data from “Region 1” when a 100 g load was positioned on the mats. The red lines (differential electrode couples 1–2, 3–4, 5–6, and 7–8) represent the pristine Kombucha, while the blue lines (differential electrode couples 9–10, 11–12, 13–14, and 15–16) represent the functionalized Kombucha. A fundamental oscillation mode occurs across all channels at 1 mHz, with visible superior harmonics at 2, 3, and 4 mHz. It is unclear whether the source of this ultralow frequency mode is an external noise disturbance or part of the mat’s electrical oscillations. Notably, there is a broad peak spanning 0.1–0.2 Hz, a genuine signal component from the functionalized mat not observable in the pristine mat data. The complexity measures display consistent behavior where the curves either show a lower plateau for the first 500 min followed by recovery to higher levels for the remaining 4000 min (SE, TE, RE, SF, KE, LZC, and DI, where SE stands for Shannon entropy, TE represents Tsallis entropy, RE stands for Rényi entropy, SF represents space filling, KE represents Kolmogorov complexity, LZC represents Lempel–Ziv complexity, and DI represents diversity index) or an initial higher plateau followed by decay to lower values (SI, PCI, and FD, where SI represents Simpson index, PCI represents permuted complexity index, and FD represents fractal dimension). This demonstrates that implementing proper HOC measures makes it feasible to interpret the mat’s spontaneous oscillations as a sensor response with long time scales.

Examining data from “Region 2”, where a 200 g load was applied for 300 min, the shorter duration limits comparability to the complexity trends in Region 1. However, noteworthy FFT responses occur, including cleaner oscillation profiles and specific modes at 4.6, 7.3, 11.6, and 14.6 mHz visible on the pristine mat. Similar modes at slightly shifted frequencies also emerge on the functionalized mat, generally skewed toward faster processes, potentially indicative of increased conductivity.

**Figure 12:**
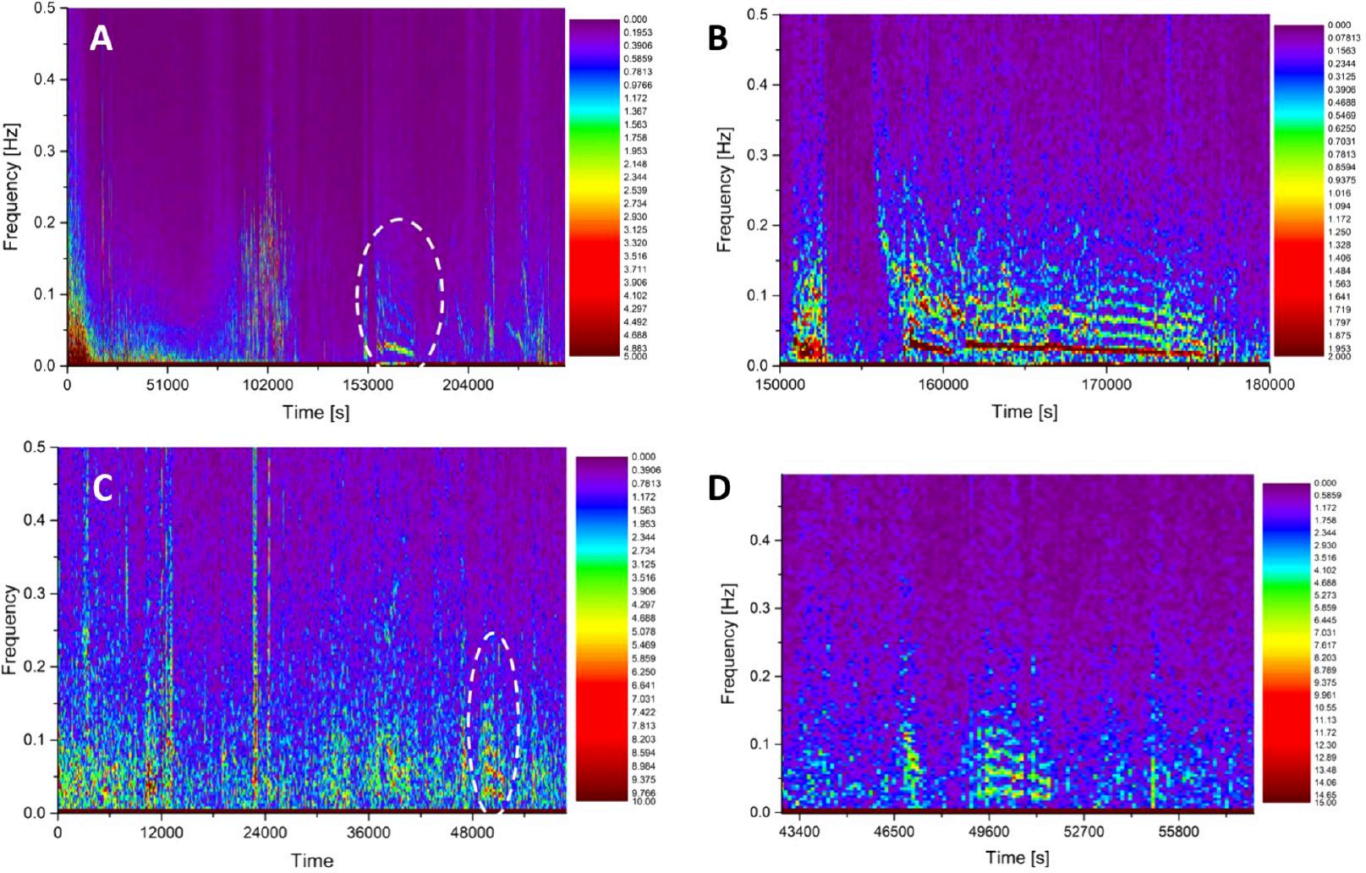
Regional details of the spiking activity features. (A) Short-time fast Fourier transforms on the region corresponding to 100 g loading of the differential channels 13–14 of the functionalized Kombucha mat. (B) A zoomed-in view highlights a peculiar feature, as indicated by the ellipse. (C) Short-time fast Fourier transforms on the region corresponding to 100 g loading of the differential channels 7–8 of the pristine Kombucha mat. (D) A zoomed-in view highlights a peculiar feature, as indicated by the ellipse.

Further analysis using short time Fourier transforms (STFT) generates spectrograms with color-coded intensity fluctuations over time and frequency, depicted in Figure 12. When a specific spectral component (vertical axis, frequencies from direct current up to 500 mHz) carries a higher energy, its fingerprint appears with a color different from violet (stretching into blue, then green, yellow and red for the most energetic harmonics). The horizontal axis is associated with time and shows the evolution of the spectrum during long measurements. The top row shows the STFT for a functionalized mat differential couple (Region 1). A specific 5 h period exhibits multiple harmonics and a complex structure circled with a dashed ellipse and magnified in panel B, resembling a vocal signal with fundamentals around 30 mHz and harmonics approaching 200 mHz. Such a complex structure is composed by quasi-parallel bands; seven of them can be easily counted, starting from the strongest (fundamental at 30 mHz, red band) and extending up to the highest harmonic (200 mHz, cyan band that is 4 times less strong than the fundamental). Interestingly, a comparable 1 h fragment occurs in Region 2 data from the pristine mat with fundamentals around 30 mHz and a still visible fifth harmonic near 150 mHz, and this time the strength drop is much less pronounced (20% less). We might say that, by analogy, the electric melody provoked by spontaneous oscillations in pristine Kombucha carries a compact sound including both lower and higher frequencies, while the one recorded in functionalized Kombucha carries a sound with a much lower timbre. Audio files were generated by the sonification of these recordings along with another excerpt from Region 3 using Melobytes software (Gardos Software Ltd. https://melobytes.com) and are provided as Supporting Information.

## Discussion

Kombucha mats are unique symbiotic systems where over 60 species of yeasts and bacteria cooperate. Pure Kombucha mats exhibit spiking activity due to the waves of depolarization traveling in the mats, emerging from metabolically triggered release of potassium.^58^ Potassium has a role in biofilm formation,^59,60^ and its release can generate changes in potential.^61^ Action potential electrical signaling is mediated by ion channels,^62^ and it is possible that the bacteria within the cellulosic matrix use potassium ion-channel-mediated electrical signals to coordinate metabolism.^61^ Moreover, the yeast community identified in the Kombucha mats has demonstrated glicolytic oscillations which are reflected in oscillations of their resistance and capacitance,^63^ possibly leading to electrical potential difference generated during the glycolysis process.^64^ Although pure Kombucha mats exhibit spiking dynamics, their electrical conductivity is limited. To improve the electrical characteristics, graphene and zeolite nanoparticles were integrated into the cellulose network. The arrangement of the material’s components, their interactions, and metabolic activity provide a unique fingerprint of electrical features and spiking activity. Two key findings are memfractive behavior and distinctive electrical spiking in response to mechanical loading. Memfractive materials combine properties of memristors, memcapacitors, and meminductors.^65^ As a memristive material implied by its material nature,^66−69^ Kombucha mats could enable various logic circuits,^70^ stateful logic operations,^71^ memory-aided logic circuits,^72^ logic operations within passive crossbar arrays of memristors,^73^ memory-aided logic circuits,^72^ self-programmable logic circuits,^74^ and memory devices.^75^ Their memfractive nature also makes the mats suitable for integration into diverse memory and computing devices, including biocompatible electronics and biowearables.

## Conclusion

In this study, we have demonstrated that Kombucha zoogleal mats, functionalized with graphene and zeolite particles, exhibit unique electrical properties. The observed electrical spiking from mechanical stimulation (i.e., applying loads) could be harnessed to create biocompatible sensors. Incorporating the mats into wearable devices or implantable sensors may facilitate the real-time detection of physiological changes in the human body or other biological systems with potential applications in real-time health monitoring. The electrical spiking could also be explored for energy harvesting purposes. If the spiking can be converted into a usable form of energy, then it might contribute to powering small electronic devices or sensors in remote or hard-to-reach locations. Combining Kombucha zoogleal mats with functional nanoparticles could lead to hybrid systems that leverage the strengths of both biological and synthetic elements.

This work demonstrates the early stage potential for hybrid systems combining Kombucha zoogleal mats with functional nanoparticles to leverage both biological and synthetic elements. This interdisciplinary approach could open up new possibilities in sustainable electronics and computing applications that are impossible with either system alone. Specifically, the biodegradable nature of Kombucha materials makes them well-suited for environmentally friendly devices and components aimed at reducing e-waste. Devices or components that naturally decompose after use could reduce electronic waste and positively impact sustainability.

## Data Availability

The data is accessible via the online database Zenodo and can be accessed at https://doi.org/10.5281/zenodo.10445665.

## Supporting Information Available

Sonification of spectra from Region 2, channels 7–8, 13–14, and 15–16, respectively (MP3, MP3, and MP3)

## Supplementary Material

ao4c01227_si_001.mp3

ao4c01227_si_002.mp3

ao4c01227_si_003.mp3
